# Positive association between omega-3/6 polyunsaturated fatty acids and idiopathic normal pressure hydrocephalus: a mendelian randomization study

**DOI:** 10.3389/fgene.2023.1269494

**Published:** 2023-12-19

**Authors:** Jilai Li, Ning Huang, Xiang Zhang, Jie Peng, Qin Huang

**Affiliations:** Department of Neurosurgery, The Second Affiliated Hospital of Chongqing Medical University, Chongqing, China

**Keywords:** idiopathic normal pressure hydrocephalus, omega-3 fatty acids, omega-6 fatty acids, mendelian randomization, causality

## Abstract

**Background:** Idiopathic normal pressure hydrocephalus (iNPH) is a common disease among the elderly, which brings great harm to the health of patients and imposes a huge economic burden on the healthcare system. Research has shown that it is possible to prevent iNPH through nutritional and dietary interventions. Intake of omega-3 and omega-6 polyunsaturated fatty acids (PUFAs) can reduce the risk of many diseases. In this study, we aimed to explore the association between omega-3/6 PUFAs and iNPH.

**Methods:** We conducted a two-sample Mendelian randomization (MR) study using summary data from publicly available genome-wide association studies (GWAS) to evaluate the potential impact of omega-3 and omega-6 PUFAs on the risk of iNPH in European populations. Inverse variance weighting was used as the main method for MR analysis, with Wald ratio, weighted median, MR-Egger, simple mode, and weighted mode as supplementary methods. In addition, we performed a series of instrument variable strength evaluations and sensitivity analyses to test the reliability of the study results. Finally, we also conducted a linkage disequilibrium score regression (LDSC) analysis to assess the genetic correlation and distinguish between causal associations and shared genetic variants between PUFAs and iNPH.

**Results:** One SD increase in genetically predicted levels of total omega-3 PUFAs (OR: 0.748; 95% CI: 0.597–0.937; *p* = 0.012; IVW), DHA (OR: 0.709; 95% CI: 0.532–0.945; *p* = 0.019; IVW), ALA (OR: 0.001; 95% CI: 1.17E-06–0.423; *p* = 0.026; Wald ratio), and DHA (OR: 0.709; 95% CI: 0.532–0.945; *p* = 0.019; IVW) were associated with a reduced iNPH risk. LDSC did not reveal any significant genetic correlations.

**Conclusion:** Higher genetically predicted levels of total omega-3 PUFAs, ALA, DHA, and DPA are associated with a reduced risk of iNPH. These findings have important implications for preventing iNPH and future nutritional guidance.

## Introduction

Idiopathic normal pressure hydrocephalus (iNPH) is a neurological disorder characterized by an enlarged ventricle in the brain with normal cerebrospinal fluid pressure. Up to date, there was no reason to explain the specific pathophysiology (Bräutigam et al., 2019). Patients often exhibit the classic triad of symptoms, including gait and balance disturbances, urinary incontinence, and cognitive impairment. Because of an age-related disease, the incidence of iNPH will double with increasing age. According to previous research reports, the incidence rate in the population aged 65 and above is approximately 3.7%, increasing to 5.9% in those aged 80 and above, and sometimes even rising to 8.9% in some areas, which is about four times of incidence rate (2.1%) in the population aged 65–79 (Jaraj et al., 2014). Clearly, iNPH patients are common in the elderly population. So far, ventriculoperitoneal shunt is the main treatment method, but the invasiveness of the surgical process and postoperative complications are emerged (Israelsson et al., 2020). According to recent research, the NPH vascular hypothesis, which has obtained important empirical evidence, suggests that prevention of iNPH may be possible through nutritional and dietary interventions (McGirr et al., 2015).

Omega-3 and omega-6 polyunsaturated fatty acids (PUFAs) play an important role in human health and various diseases (Kapoor et al., 2021). They can not be synthesized endogenously by the human body but can only be obtained from the diet. As a result, they are also called essential fatty acids. Omega-3 PUFAs include alpha-linolenic acid (ALA), eicosapentaenoic acid (EPA), docosapentaenoic acid (DPA), and docosahexaenoic acid (DHA). They can be directly obtained from fatty fish or seaweed. Meanwhile, EPA, DPA, and DHA can also be indirectly supplemented by consuming plant oils rich in ALA, then converted into EPA, DPA, and DHA (Dyall et al., 2022). Linoleic acid (LA) belongs to omega-6 PUFAs, which are mainly gained from consuming vegetable oils and converted into arachidonic acid (AA) in the human body (Dyall, 2017). In the past few decades, the effects of PUFAs on inhibiting inflammation, reducing obesity, lowering blood pressure, preventing cardiovascular diseases and autoimmune diseases have been effectively verified (Russo, 2009; Marion-Letellier et al., 2015; Li et al., 2019). Inflammatory reactions and high blood pressure play important roles in the occurrence or development of iNPH (Wang et al., 2020). Therefore, in theory, PUFAs may have the potential to reduce the incidence and delay the progression of iNPH. Unfortunately, there have been no relevant studies on this so far.

To fill this gap, we applied Mendelian randomization (MR) on the basis of genome-wide association studies (GWAS) data to evaluate the potential relationship between omega-3 and omega-6 PUFAs and the risk of iNPH. The MR method is an epidemiological approach that uses genetic variations as instrumental variables (IVs) to infer underlying associations between exposures and outcomes (Davies et al., 2018). Because genetic variation is randomly assigned to a given allele during conception (similar to a randomized trial) and remains constant after conception, the MR study can overcome the influence of confounding factors and reverse causality on causal inference (Smith and Ebrahim, 2003). In addition, to determine if MR findings could be influenced by genetic similarity, we conducted linkage disequilibrium (LD) score regression (LDSC) analysis (Bulik-Sullivan et al., 2015a).

## Methods

### Study design

**FIGURE 1:**
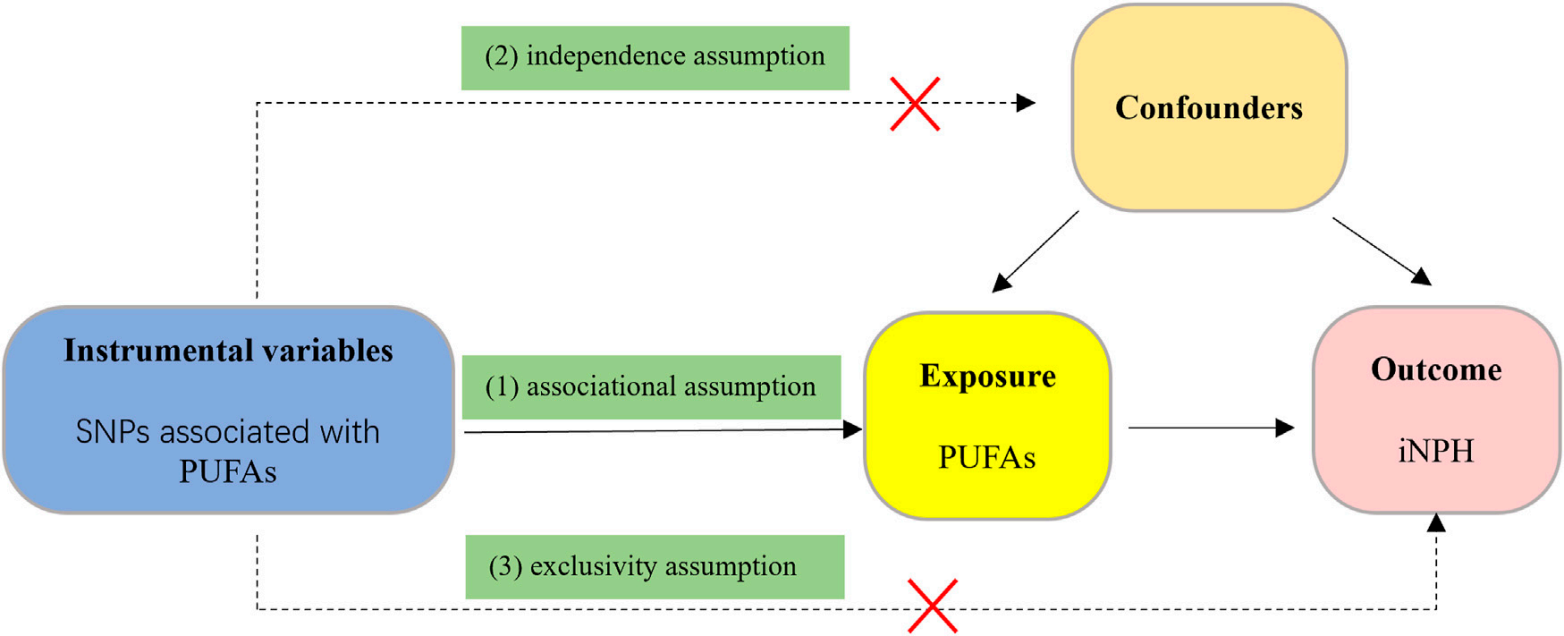
The flow diagram of the MR study. (1) The genetic instrumental variables (IVs) are strongly associated with PUFAs (associational assumption); (2) The genetic IVs do not affect the outcome through the confounders (independence assumption); (3) The genetic IVs do not affect iNPH directly, but only via indirect exposure (exclusivity assumption).

We employed a two-sample MR design in this study. As publicly available research data from genome-wide association studies (GWAS) were used, which have been approved by relevant institutional review boards, no additional informed consent or ethical approval was required. The three core assumptions of effective Mendelian randomization (MR) analysis are: 1) The genetic instrumental variables (IVs) are strongly associated with PUFAs (associational assumption); 2) The genetic IVs do not affect the outcome through the confounders (independence assumption); 3) The genetic IVs do not affect iNPH directly, but only via indirect exposure (exclusivity assumption) (Burgess et al., 2019). the overview of the study design is shown in Figure 1.

### Data sources for PUFAs

In order to ensure high effectiveness and full repeatability of our MR analysis, our study collected publicly available GWAS summary data. First, we extracted single-nucleotide polymorphisms (SNPs) related to total omega −3 PUFAs and total omega-6 PUFAs from the latest GWAS on circulating PUFAs, which included 114,999 individuals of European ancestry and can be accessed at https://gwas.mrcieu.ac.uk/. Summary-level data related to specific types of omega-3 and omega-6 PUFAs mentioned above could also be obtained from the CHARGE (Cohorts for Heart and Aging Research in Genomic Epidemiology) consortium. Among them, data on the summary level of AA came from a meta-analysis of a GWAS by the CHARGE consortium in 2014, which included 8,631 Caucasian adults from five prospective studies (Guan et al., 2014). Data on the summarized levels of ALA, DPA, and EPA came from a meta-analysis of a GWAS in 2011, including 8,866 participants of European ancestry from five population-based cohort studies (Lemaitre et al., 2011). In addition, data on the summarized levels of DHA and LA are both from a large-scale GWAS in the UK Biobank. For more detailed information regarding these GWAS samples used in this analysis, please refer to Table 1.

**TABLE 1:** Sources and sample size of genetic IVS for exposures and outcome.

Traits	SNPs	Data sources	Sample size (case/control)	Ancestry
ω-3 PUFAs	46	UK Biobank	114,999	European
DHA	41	UK Biobank	114,999	European
DPA	3	CHARGE	8,866	European
EPA	4	CHARGE	8,866	European
ALA	1	CHARGE	8,866	European
ω-6 PUFAs	50	UK Biobank	114,999	European
LA	43	UK Biobank	114,999	European
AA	93	CHARGE	8,631	European
iNPH	-	FinnGen	767/376,377	European

### Data sources for iNPH

The GWAS data for iNPH were based on the ninth round of FinnGen research released in 2023. The FinnGen study is an unprecedented global research project launched in Finland in the fall of 2017 that combines genomic information with digital healthcare data (Kurki et al., 2023). The ninth round of the FinnGen study involved 767 patients defined as NPH and 350,251 controls, all of whom had European ancestry. The diagnosis of NPH is based on the International Classification of Diseases, 10th Revision (ICD-10) standard, with the code G91.2. Details of the INPH outcome GWAS meta-analysis are described in Table 1.

In this study, there was no significant sample overlap between the exposure and outcome groups in the summary data of genome-wide association studies (GWAS). This suggests that our results are more reliable and accurate. Additionally, both the exposure and outcome groups had European ancestry, which is more closely related genetically, helping to reduce biases due to differences in genetic background. Therefore, we can have more confidence in interpreting and using these research findings.

### Two-sample mendelian randomization

#### IV selection

The screening criteria for SNPs include significant genome-wide association between the instrumental variable SNPs and the corresponding exposure (P < 5E-08), as well as no linkage disequilibrium block between the instrumental variable SNPs and the corresponding outcome data (r2 = 0.001, strand alignment = 10,000 kb) (Burgess and Thompson, 2011). In addition, if instrumental SNPs are not found in the outcome data or if they have a palindromic sequence, they will also be removed. At the same time, the F-statistic of SNPs is calculated to estimate the strength of IVs, meeting the first MR assumption (Burgess and Thompson, 2011).

#### Statistical analysis

We harmonized the statistical data for SNP-PUFAs and SNP-iNPH to ensure that the allele frequencies for each SNP were consistent between PUFAs and iNPH. Six Mendelian randomization (MR) methods were employed: Wald ratio, inverse-variance weighted (IVW), MR-Egger, weighted median, weighted mode and simple mode. If only one SNP was used as the genetic instrumental variable, we performed MR analysis using the Wald ratio method (Bautista et al., 2006). The Wald ratio method (i.e., the beta coefficient for the SNP’s effect on the outcome divided by the beta coefficient for the SNP’s effect on the exposure) was used to infer causal relationships between exposure and outcomes with only one SNP as the genetic instrumental variable (Bautista et al., 2006). For multiple SNPs as genetic instrumental variables, we used other methods for MR analysis. IVW is the traditional standard method for summarizing data in MR, which can directly calculate the size of the causal effect using summary data without requiring individual-level data (Lawlor et al., 2008). It uses inverse variance as weights, giving smaller weights to SNPs with larger standard errors when combining effect estimates, thereby reducing their impact on the overall result. The IVW method can be used not only for binary outcome analysis but also for continuous outcome analysis, making it highly versatile. Median estimation includes weighted median, simple mode, and weighted mode. The difference between the weighted median, simple mode and weighted mode lies in the way they consider the weights and frequencies of the data. Weighted median considers the weight of each data point, simple mode only considers the most frequently occurring data, while weighted mode considers both weight and frequency. Using the weighted median method to estimate the effect allows us to find the weighted empirical distribution function of all selected SNPs. By using the weighted median method, SNPs with stronger effects contribute more to the causal estimate, and the bias is smaller when fewer SNPs are effective instruments (Bowden et al., 2016). The basic idea of MR-Egger is to use Egger regression to fit a linear regression model on the effect estimates of all SNPs in order to estimate the true causal effect and test for the presence of genetic directional pleiotropy bias (Bowden et al., 2015). The MR-Egger method allows for a certain degree of bias in the influence of genotypes on outcomes, making it more accurate and reliable in exploring causal relationships (Lin et al., 2022).

#### Sensitivity analysis

To ensure the robustness of our results, we conducted a series of sensitivity analyses. The MR-PRESSO method detects the presence of pleiotropy by testing for correlation between genotypes and outcomes, and corrects for bias caused by correlation between genotypes and outcomes using regression methods (Verbanck et al., 2018). The MR-PRESSO method can also detect and correct for the influence of outliers on MR estimation results, thereby improving the accuracy and reliability (Bowden et al., 2019). In addition, in heterogeneity testing of MR results, MR-Egger and IVW methods also play important roles. The MR-Egger regression method can not only detect the heterogeneity of the results but also provide an MR-Egger intercept, which can be used to assess the presence of pleiotropic effects (Burgess and Thompson, 2017). In the leave-one-out analysis, we will remove each individual genetic variant used in the MR analysis one by one, and then recalculate the MR effect estimate to evaluate the influence of each variant on the final effect estimate. Finally, to better display and understand the results of MR analysis, we plotted leave-one-out analysis plots, forest plots, scatter plots, and funnel plots based on the results of MR analysis. All results were presented as the odds ratio (OR) with their 95% confidence interval (CI), and *p* < 0.05 was considered statistically significant. All statistical analyses were performed using R version 4.2.1 and the MR packages (TwoSampleMR and MR-PRESSO) (Yavorska and Burgess, 2017). The power calculation was performed using an online power calculator (mRnd) (https://cnsgenomics.com/shiny/mRnd/) (Brion et al., 2013).

We used LDSC to assess overall genetic correlation in order to investigate whether the clear associations between PUFAs and iNPH are due to shared causal genetic variants (Bulik-Sullivan et al., 2015a). LDSC is an advanced method that does not require individual genotypes, genome-wide significant SNPs, or LD pruning (which can result in loss of information if the causal SNP is located in LD), but instead uses all available SNPs. In addition, this approach can greatly reduce biases due to sample overlap. Following the publicly released LDSC protocol (available from https://github.com/bulik/ldsc), we used the LD-score regression software in Python to estimate genetic correlation (Bulik-Sullivan et al., 2015b; Zheng et al., 2017). The resulting estimates represent the genetic covariance between GWA traits based on all polygenic effects captured by SNPs.

## Results

### IV selection

Ultimately, there are 46 independent SNPs for total omega-3 PUFAs, 50 independent SNPs for total omega-6 PUFAs, 1 SNP for ALA, 3 SNPs for DPA, 41 SNPs for DHA, 4 SNPs for EPA, 93 SNPs for AA, and 43 SNPs for LA as instrumental variables (Supplementary Table). The F-statistics for all the instruments are greater than 10, which helps to effectively eliminate the bias from weak instruments (Freeman et al., 2013). The instrument strength and MR power are available in Supplementary Tables.

### Causal effects of PUFAs on iNPH

**FIGURE 2:**
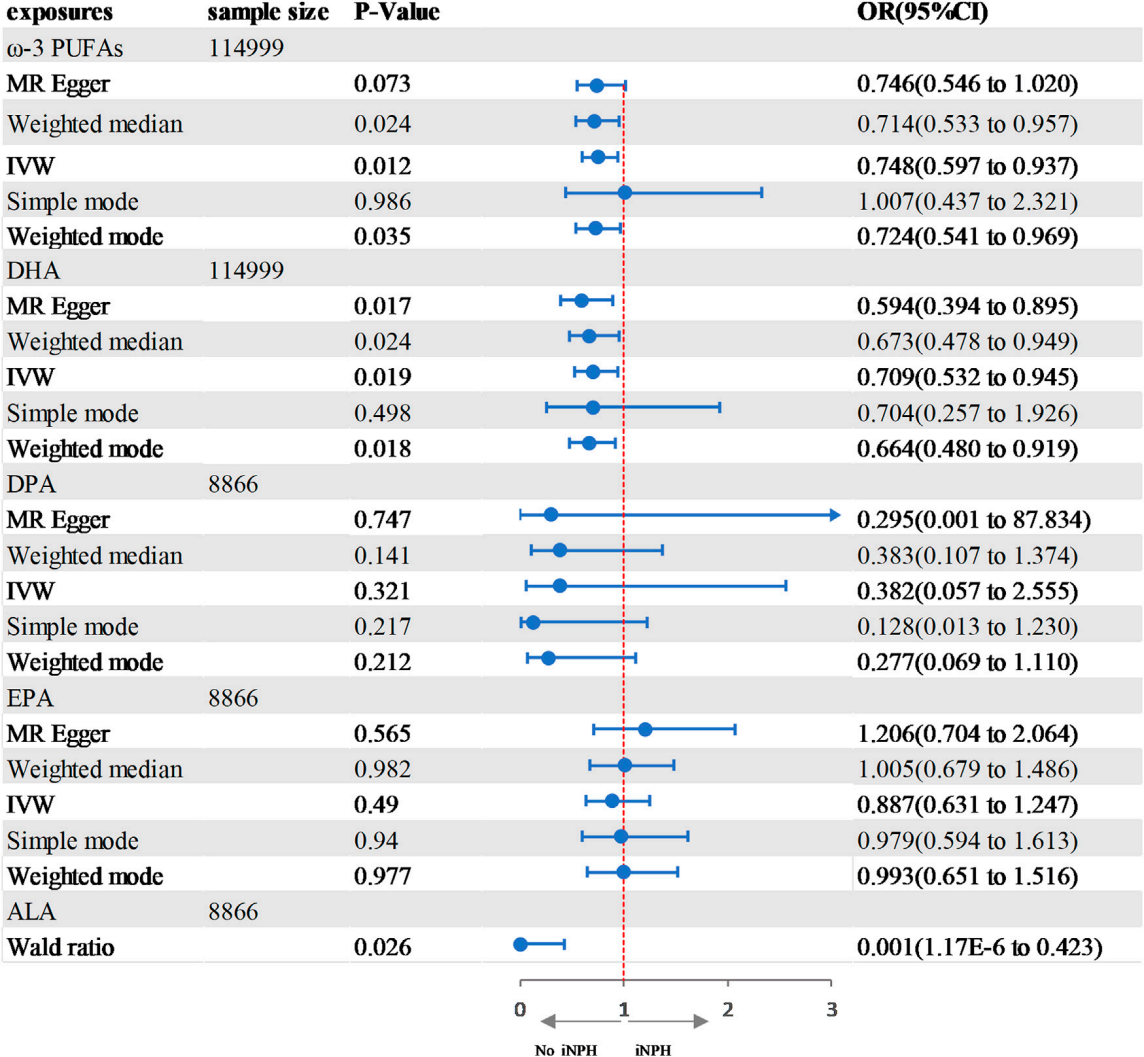
Forest plot of the genetic associations between ω-3 PUFAs and iNPH.CI, confidence interval; IVW, inverse variance-weighted; MR, Mendelian randomization; OR, odds ratio.

**FIGURE 3:**
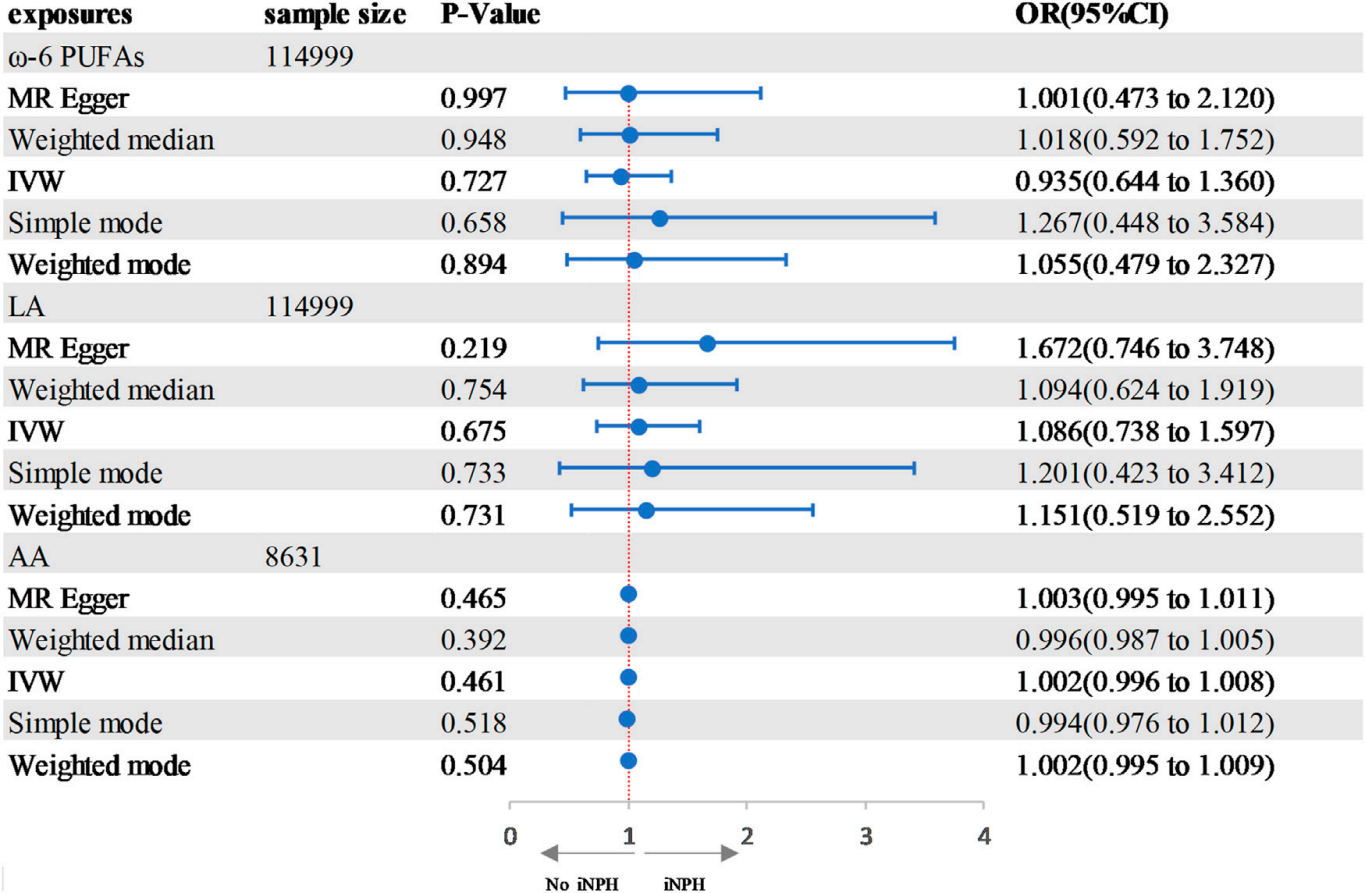
Forest plot of the genetic associations between DHA and iNPH. CI, confidence interval; IVW, inverse variance-weighted; MR, Mendelian randomization; OR, odds ratio.

**FIGURE 4:**
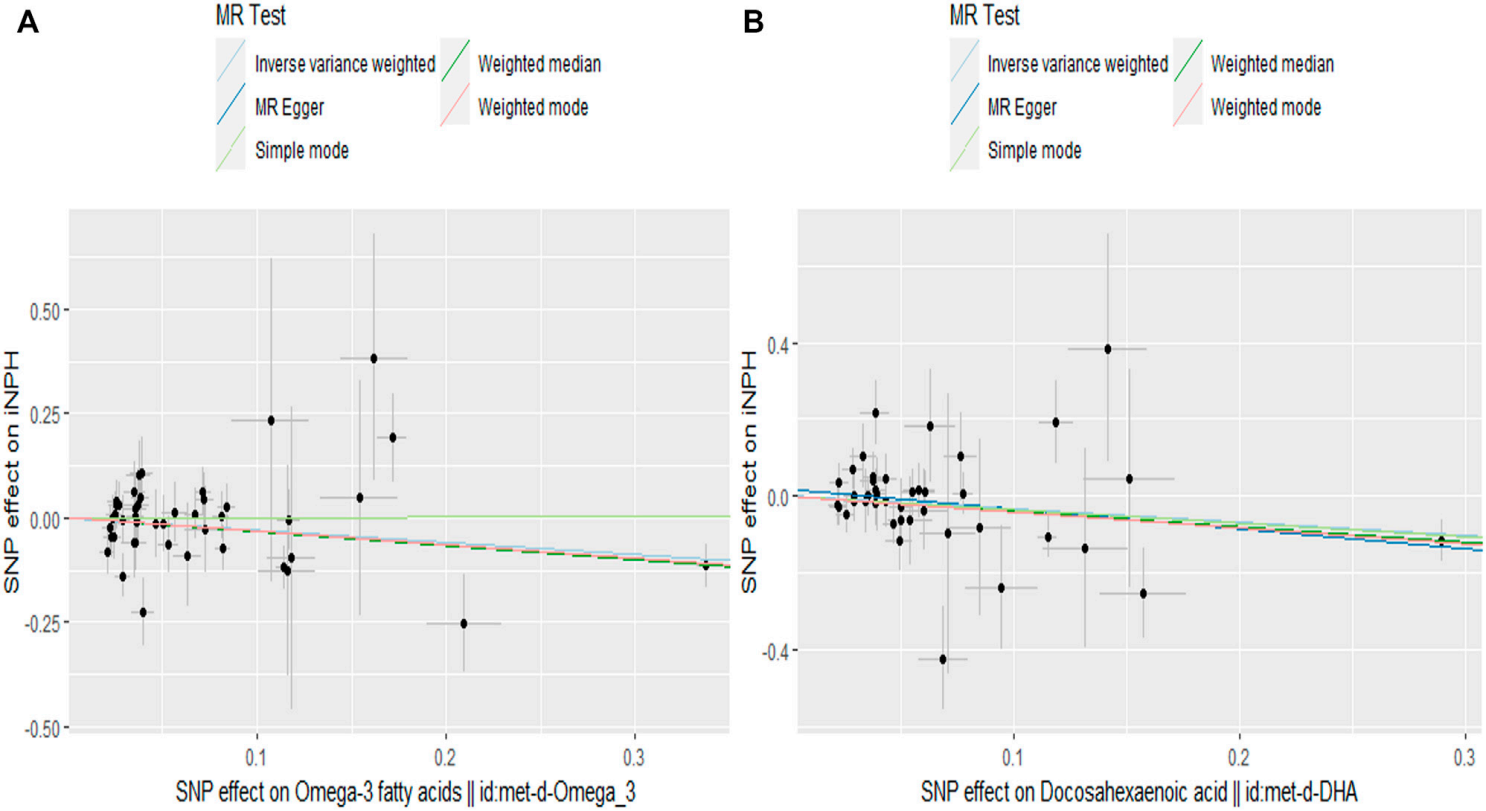
Scatter plot of the effects of SNPs on PUFAs and iNPH. The horizontal and vertical axes represent the effect of each genetic variation on **(A)** ω-3 PUFAs **(B)** DHA and iNPH. The gray line around the black solid point indicates the corresponding 95% CI for the effect. The slopes of the solid lines show the effect estimates of the 5 MR methods. MR, Mendelian randomization; SNP, single nucleotide polymorphism.

In the two-sample MR analysis, we evaluated the potential effects of PUFAs on the development of INPH, including omega-3 PUFAs, omega-6 PUFAs, and some specific types of them. Scatter plots are shown in Supplementary Figures. The results are presented in Table 2 and Figure 2 and Figure 3. According to the random-effects IVW analysis, one standard deviation (SD) increase in genetically predicted levels of total omega-3 fatty acids (OR: 0.748; 95% CI: 0.597–0.937; *p* = 0.012; IVW), DHA (OR: 0.709; 95% CI: 0.532–0.945; *p* = 0.019; IVW) and ALA (OR: 0.001; 95% CI: 1.17E-06–0.423; *p* = 0.026; Wald ratio) were associated with a decreased risk of iNPH per unit increase. Scatter plot (Figure 4) also indicates a negative correlation between total omega-3 PUFAs and DHA with iNPH, while Scatter plots of genetic associations between other PUFAs and iNPH are shown in Supplementary Figures. ALA (OR: 0.001; 95% CI: 1.17E-06–0.423; *p* = 0.026; Wald ratio) was favorably with iNPH. Moreover, DPA (OR: 0.382; 95% CI: 0.057–2.555; *p* = 0.321; IVW), EPA (OR: 0.887; 95% CI: 0.631–1.247; *p* = 0.490; IVW) and omega-6 PUFAs, including total omega-6 PUFAs (OR: 0.935; 95% CI: 0.644–1.360; *p* = 0.727; IVW), LA (OR: 1.086; 95% CI: 0.738–1.597; *p* = 0.675; IVW) and AA (OR: 1.002; 95% CI: 0.996–1.008; *p* = 0.461; IVW) were found to be unrelated to iNPH.

**TABLE 2:** The MR results of the relationship between PUFAs and iNPH.

Exposures	Method	SNP	Beta	SE	OR	OR_lci95	OR_uci95	*p*
**ω-3 PUFAs**	MR Egger	46	−0.293	0.159	0.746	0.546	1.020	0.073
	Weighted median	46	−0.337	0.149	0.714	0.533	0.957	0.024
	IVW	46	−0.290	0.115	0.748	0.597	0.937	0.012
	Simple mode	46	0.007	0.426	1.007	0.437	2.321	0.986
	Weighted mode	46	−0.323	0.149	0.724	0.541	0.969	0.035
**DHA**	MR Egger	41	−0.521	0.209	0.594	0.394	0.895	0.017
	Weighted median	41	−0.396	0.175	0.673	0.478	0.949	0.024
	IVW	41	−0.343	0.146	0.709	0.532	0.945	0.019
	Simple mode	41	−0.351	0.513	0.704	0.257	1.926	0.498
	Weighted mode	41	−0.409	0.166	0.664	0.480	0.919	0.018
**DPA**	MR Egger	3	−1.221	2.907	0.295	0.001	87.834	0.747
	Weighted median	3	−0.959	0.651	0.383	0.107	1.374	0.141
	IVW	3	−0.961	0.969	0.382	0.057	2.555	0.321
	Simple mode	3	−2.057	1.155	0.128	0.013	1.230	0.217
	Weighted mode	3	−1.284	0.708	0.277	0.069	1.110	0.212
**EPA**	MR Egger	4	0.187	0.274	1.206	0.704	2.064	0.565
	Weighted median	4	0.005	0.200	1.005	0.679	1.486	0.982
	IVW	4	−0.120	0.174	0.887	0.631	1.247	0.490
	Simple mode	4	−0.021	0.255	0.979	0.594	1.613	0.940
	Weighted mode	4	−0.007	0.216	0.993	0.651	1.516	0.977
**ALA**	Wald ratio	1	−7.260	3.265	0.001	1.17E-06	0.423	0.026
**ω-6 PUFAs**	MR Egger	50	0.001	0.383	1.001	0.473	2.120	0.997
	Weighted median	50	0.018	0.277	1.018	0.592	1.752	0.948
	IVW	50	−0.067	0.191	0.935	0.644	1.360	0.727
	Simple mode	50	0.236	0.531	1.267	0.448	3.584	0.658
	Weighted mode	50	0.054	0.403	1.055	0.479	2.327	0.894
**LA**	MR Egger	43	0.514	0.412	1.672	0.746	3.748	0.219
	Weighted median	43	0.090	0.287	1.094	0.624	1.919	0.754
	IVW	43	0.082	0.197	1.086	0.738	1.597	0.675
	Simple mode	43	0.183	0.533	1.201	0.423	3.412	0.733
	Weighted mode	43	0.141	0.406	1.151	0.519	2.552	0.731
**AA**	MR Egger	93	0.003	0.004	1.003	0.995	1.011	0.465
	Weighted median	93	−0.004	0.005	0.996	0.987	1.005	0.392
	IVW	93	0.002	0.003	1.002	0.996	1.008	0.461
	Simple mode	93	−0.006	0.009	0.994	0.976	1.012	0.518
	Weighted mode	93	0.002	0.004	1.002	0.995	1.009	0.504

### Results of sensitivity analysis

Pleiotropy and heterogeneity test were also shown in Table 3. The MR-PRESSO global test did not observe any outliers or horizontal pleiotropy in the MR analysis results. In addition, two methods, IVW and MR-Egger, were used to detect heterogeneity, and there was no evidence of heterogeneity. Meanwhile, MR-Egger regression can also be used to analyze the horizontal pleiotropy of genes. Based on the intercept value of the MR-Egger regression, no horizontal pleiotropy was detected for the causal effect of PUFAs on hydrocephalus. A leave-one-out stability test was conducted by excluding one SNP at a time (see Supplementary Figure). After excluding one SNP at a time, the potential association estimates between genetic predictions of various types of PUFAs and the risk of iNPH did not change significantly, indicating that the potential driving SNP is unlikely to cause bias in the causal relationship. Funnel plots are listed in Supplementary Figures.

**TABLE 3:** Pleiotropy and heterogeneity test of the PUFAs IVs from iNPH GWAS.

Exposures	Heterogeneity				Pleiotropy		
	Method	Q	Q_df	*p*	Intercept	SE	*p*
ω-3 PUFAs	MR Egger	44.963	44	0.431	0.000	0.014	0.982
	IVW	44.964	45	0.473			
DHA	MR Egger	44.919	39	0.238	0.020	0.017	0.243
	IVW	46.537	40	0.221			
DPA	MR Egger	5.167	1	0.023	0.015	0.146	0.936
	IVW	5.220	2	0.074			
EPA	MR Egger	0.055	2	0.973	−0.095	0.066	0.285
	IVW	2.149	3	0.542			
ALA	-	-	-	-	-	-	-
ω-6 PUFAs	MR Egger	62.774	48	0.075	−0.004	0.021	0.838
	IVW	62.830	49	0.089			
LA	MR Egger	51.594	41	0.124	−0.028	0.024	0.240
	IVW	53.380	42	0.112			
AA	MR Egger	66.735	91	0.974	−0.004	0.015	0.797
	IVW	66.801	92	0.978			

### Results of LDSC

To investigate alternative explanations for the shared genetic components, we conducted LDSC to examine whether the genetic associations behind PUFAs and iNPH could be due to shared causal genetic variants (Table 4). We did not find significant genetic correlations between them (rg = 0.002 and *p* = 0.984 between total omega-3 PUFAs and iNPH; rg = 0.006 an *p* = 0.947 between DHA and iNPH; and rg = 0.354 and *p* = 0.328 between DPA and iNPH; and rg = 0.003 and *p* = 0.972 between total omega-6 PUFAs and iNPH; and rg = 0.004 and *p* = 0.966 between LA and iNPH; and rg = 0.071 and *p* = 0.895 between AA and iNPH). However, due to small sample size or low heritability, we were unable to complete the LDSC analysis between EPA, ALA, and iNPH.

**TABLE 4:** Genetic correlations between PUFAs and iNPH by LD score regression (LDSC).

Trait	rg	SE_rg	*p*_rg	h2_observed	h2_observed_se	h2_int	h2_Z	h2_p
W3	0.002	0.085	0.984	18.623	0.391	14.952	47.636	<0.001
DHA	0.006	0.087	0.947	18.604	0.387	15.105	48.065	<0.001
DPA	0.354	0.361	0.328	0.055	0.051	1.013	1.08	0.28
EPA	-	-	-	-	-	-	-	-
ALA	-	-	-	-	-	-	-	-
W6	0.003	0.086	0.972	18.646	0.386	15.005	48.301	<0.001
LA	0.205	0.1	0.041	0.102	0.014	1.026	7.48	<0.001
AA	0.071	0.535	0.895	0.028	0.059	1.021	0.472	0.637

rg = genetic correlation; SE_rg = standard error of rg; *P*_rg = *p*-value for rg. h2_int = heritability intercept; h2_Z = Z score of heritability; h2_p = *p*-value of heritability.

## Discussion

To our knowledge, this is the first large-scale MR study to evaluate the relationship between PUFAs and iNPH. Based on the summary data of GWAS, we used various statistical methods to evaluate the causal relationship between many types of PUFAs and iNPH. We found that omega-3 PUFAs (including DHA and ALA) were significantly negatively associated with iNPH. For other exposure factors such as EPA, DPA and omega-6 PUFAs (including LA and AA), there was not enough genetic evidence to demonstrate any potential relationship between them and iNPH. These results suggested that higher levels of total omega-3 PUFAs, ALA, and DHA can significantly reduce the risk of iNPH in the population, indicating that they are potential protective factors for iNPH. Despite the lack of evidence for genetic correlation between PUFAs and iNPH from the LDSC analysis, a series of sensitivity analysis results confirmed that the above findings are reliable and robust.

To date, no authoritative studies on the association between PUFAs levels and the risk of iNPH have been published, and it is still unknown whether PUFAs can reduce the risk of iNPH. However, our MR study results found that high levels of omega-3 PUFAs (including ALA and DHA) could effectively decrease the risk of iNPH in the population. The reasons for this may be as follows.

It is widely recognized that changes in cerebrospinal fluid (CSF) dynamics are the initiating factor of iNPH (Wang et al., 2020). In 1965, Hakim and Adams proposed the hypothesis that iNPH was caused by an increase in CSF-volume and compensatory ventricular enlargement (Hakim and Adams, 1965). Some research results have shown that iNPH patients have significantly reduced white matter volume, while cerebrospinal fluid volume, cerebrospinal fluid ratio, and total intracranial volume are significantly increased, supporting this hypothesis (Li et al., 2023). Currently, the main causes of CSF-volume increase are believed to be CSF absorption disorders and increased CSF production (Wang et al., 2020). First, according to the theory of reduced cerebral blood flow, CSF reflux obstruction is often caused by decreased blood flow to small vessels in the deep white matter of the brain (Kristensen et al., 1996; Chang et al., 2009). Research has shown that omega-3 PUFAs can improve endothelial dysfunction by reducing the production of inflammatory cytokines and promoting the release of nitric oxide to increase endothelial dependent vasodilation, which may be one of the reasons why omega-3 PUFAs can reduce the risk of iNPH (Schini et al., 1993; He et al., 2009). In addition, according to the pulsatile vector theory, CSF circulation is caused by blood flow pulsations (Preuss et al., 2013). The blood flow pulsations generated by arterial and venous blood flow can be divided into three vector forces: interstitial fluid impulsive force, subarachnoid CSF impulsive force, and centrifugal brain expansion force. When these three vector forces reach equilibrium, cerebrospinal fluid can be reabsorbed into the venous system across the blood-brain barrier. This theory implies that an increase in interstitial fluid impulsive force is a potential cause of iNPH. Risk factors for arterial atherosclerosis such as obesity and hypertension can cause an increase in arterial blood flow pressure, which is directly proportional to interstitial fluid impulsive force (Israelsson et al., 2017; Lechner et al., 2020). However, numerous research results have shown that omega-3 PUFAs reduce arterial blood flow pressure by decreasing triglycerides, low-density lipoprotein (LDL), and very low-density lipoprotein (VLDL), increasing high-density lipoprotein (HDL), directly activating the large-conductance Ca^2^⁺-dependent K⁺ channel and improving arterial endothelial function and elasticity. This can ultimately reduce interstitial fluid impulsive force and play a beneficial role in reducing the risk of iNPH occurrence (Harris and Bulchandani, 2006; Hoshi et al., 2013; Chen et al., 2019).

In the occurrence and development of iNPH, pro-inflammatory factors produced by microglia due to ischemia and hypoxia and accumulation of toxic substances produced by cell metabolism due to damage to the glymphatic system play crucial roles (Castañeyra-Ruiz et al., 2016; Huang et al., 2021). Microglia are important immune cells in the brain, and when ischemia and hypoxia occur, microglia are activated and proliferate, releasing neurotoxic substances (especially amyloid beta and tau proteins) and inflammatory cytokines (such as TNF-α, interleukin-1β, *etc.*) to rapidly mount an immune response (Sosvorova et al., 2014). These inflammatory factors and toxic substances further damage oligodendrocytes and neuronal axons, leading to a vicious cycle. Additionally, due to glymphatic system damage in iNPH patients, there is an obstacle to the clearance of toxic substances, resulting in further accumulation of toxic substances (Tan et al., 2021). Research has found that omega-3 PUFAs can regulate the body’s inflammatory response and immune function through various mechanisms (Calder, 2015; Calder, 2017). First, omega-3 PUFAs significantly increase the content of EPA and DHA in immune cell membrane phospholipids, competitively reducing the content of AA, and omega-3 PUFAs (EPA, DHA) can competitively inhibit the oxidation of AA by cyclooxygenase, reducing the generation of AA products, which helps to inhibit inflammation and immune response. Second, omega-3 PUFAs incorporated into the lipid bilayer of lymphocytes and endothelial cells can significantly alter the composition of their membranes, affecting membrane fluidity and the spatial conformation of membrane receptors, thereby affecting the synthesis of functional molecules and cell function. In addition, omega-3 PUFAs inhibit the body’s immune response by affecting signal transduction and cytokine expression. Furthermore, omega-3 PUFAs improve cerebrovascular regulation by reducing inflammatory responses and increasing the number of perivascular macrophages, thereby enhancing the clearance capacity of the glymphatic system (Liu et al., 2020).

Our study has several strengths (Smith and Ebrahim, 2004). First, by using genetic variations as surrogate markers for PUFAs and iNPH, potential confounding and reverse causality can be reduced. Second, the IVs of exposure and outcome are both derived from existing large-scale GWAS, which makes the assessment of effect sizes more accurate than results from individual-level data or studies with limited sample sizes. Finally, in addition to the primary IVW method, we also used supplementary methods such as MR-Egger, Wald ratio, and so on. Moreover, multiple sensitivity analysis methods were performed to validate the results.

Of course, we should also note that our study has certain limitations. First, although the iNPH GWAS dataset was sourced from FinnGen and represented a relatively isolated population, which can effectively reduce the impact of sample overlap, both the exposures and outcome GWAS datasets were primarily derived from individuals of European ancestry, with very similar genetic backgrounds, which can greatly reduce biases caused by population stratification; however, caution should be exercised when applying our findings to other populations, as their reliability may be affected. Second, for some PUFAs, we only used 1-3 SNPs as instrumental variables. Therefore, regression estimates, such as the multi-effectiveness test of MR-Egger regression, may not be robust. Third, we used the latest and largest iNPH GWAS dataset from FinnGen (including 767 cases and 375,610 controls). Although we tried our best to avoid potential low power issues, they may still occur. To address this issue, we used the mRnd online tool to calculate the MR power. Although some PUFAs had low statistical power, we found that there was 100% statistical power between DPA, ALA and iNPH. However, in order to further enhance the reliability of our research results, it is necessary for us to use a larger GWAS dataset for MR analysis, which will be the focus of our future research. Fourth, the potential biological mechanisms underlying the relationship between omega-3 PUFAs and the risk of hydrocephalus remain unclear, and the MR method can only make preliminary judgments about their potential associations. Fifth, we cannot exclude the possibility of secondary NPH cases among the NPH cases studied in this research. The conclusions drawn from secondary NPH and idiopathic NPH may differ in their application. Therefore, when we emphasize iNPH, there may be bias in the results. Sixth, the LDSC analysis did not reveal substantial genetic correlations. This could be attributed to false-negative results due to either a small sample size in the GWAS dataset or weak associations between genes and phenotypes. Finally, in practical applications, it is difficult to fully satisfy the three key assumptions of MR studies, which may lead to bias in causal inference. Fortunately, a series of sensitivity analyses, including MR-Egger regression, MR-PRESSO and leave-one-out analysis, were conducted in our MR study, and no obvious heterogeneity or horizontal pleiotropy was found.

## Conclusion

Overall, although the LDSC analysis did not find substantial genetic correlations, our MR analysis provided strong genetic evidence suggesting that ω-3 polyunsaturated fatty acids (particularly DHA and ALA) play a significant role in reducing the risk of iNPH. However, EPA, DPA and omega-6 PUFAs (including LA and AA) had no apparent effect on iNPH. These findings have important implications for preventing iNPH and future nutritional guidance. In addition, further studies and researches are needed to elucidate the potential mechanisms underlying the relationship between omega-3 PUFAs and iNPH.

## Data Availability

The original contributions presented in the study are included in the article/Supplementary Material, further inquiries can be directed to the corresponding author.

## Abbreviations

AA, arachidonic acid; ALA, alpha-linolenic acid; CI, confidence interval; CSF, change in cerebrospinal fluid; DPA, docosapentaenoic acid; DHA, docosahexaenoic acid; EPA, eicosapentaenoic acid; GWAS, genome-wide association studies; HDL, high-density lipoprotein; iNPH, idiopathic normal pressure hydrocephalus; IV, instrumental variable; IVW, inverse variance weighted; LA, linoleic acid; LDL, low-density lipoprotein; LDSC, linkage disequilibrium (LD) score regression; MR, Mendelian randomization; OR, 0dds ratio; PUFAs, polyunsaturated fatty acids; SD, standard deviation; SNP: single-nucleotide polymorphism.

## References

[B1] Bautista L. E. Smeeth L. Hingorani A. D. Casas J. P. (2006). Estimation of bias in nongenetic observational studies using "mendelian triangulation. Ann. Epidemiol. 16, 675–680. 10.1016/j.annepidem.2006.02.001 16621596

[B2] Bowden J. Davey Smith G. Burgess S. (2015). Mendelian randomization with invalid instruments: effect estimation and bias detection through Egger regression. Int. J. Epidemiol. 44, 512–525. 10.1093/ije/dyv080 26050253 PMC4469799

[B3] Bowden J. Davey Smith G. Haycock P. C. Burgess S. (2016). Consistent estimation in mendelian randomization with some invalid instruments using a weighted median estimator. Genet. Epidemiol. 40, 304–314. 10.1002/gepi.21965 27061298 PMC4849733

[B4] Bowden J. Del Greco M. F. Minelli C. Zhao Q. Lawlor D. A. Sheehan N. A. (2019). Improving the accuracy of two-sample summary-data Mendelian randomization: moving beyond the NOME assumption. Int. J. Epidemiol. 48, 728–742. 10.1093/ije/dyy258 30561657 PMC6659376

[B5] Bräutigam K. Vakis A. Tsitsipanis C. (2019). Pathogenesis of idiopathic normal pressure hydrocephalus: a review of knowledge. J. Clin. Neurosci. official J. Neurosurg. Soc. Australasia 61, 10–13. 10.1016/j.jocn.2018.10.147 30409528

[B6] Brion M. J. Shakhbazov K. Visscher P. M. (2013). Calculating statistical power in Mendelian randomization studies. Int. J. Epidemiol. 42, 1497–1501. 10.1093/ije/dyt179 24159078 PMC3807619

[B7] Bulik-Sullivan B. Finucane H. K. Anttila V. Gusev A. Day F. R. Loh P. R. (2015a). An atlas of genetic correlations across human diseases and traits. Nat. Genet. 47, 1236–1241. 10.1038/ng.3406 26414676 PMC4797329

[B8] Bulik-Sullivan B. K. Loh P. R. Finucane H. K. Ripke S. Yang J. Patterson N. (2015b). LD Score regression distinguishes confounding from polygenicity in genome-wide association studies. Nat. Genet. 47, 291–295. 10.1038/ng.3211 25642630 PMC4495769

[B9] Burgess S. Davey Smith G. Davies N. M. Dudbridge F. Gill D. Glymour M. M. (2019). Guidelines for performing Mendelian randomization investigations. Wellcome open Res. 4, 186. 10.12688/wellcomeopenres.15555.2 32760811 PMC7384151

[B10] Burgess S. Thompson S. G. (2011). Avoiding bias from weak instruments in Mendelian randomization studies. Int. J. Epidemiol. 40, 755–764. 10.1093/ije/dyr036 21414999

[B11] Burgess S. Thompson S. G. (2017). Interpreting findings from Mendelian randomization using the MR-Egger method. Eur. J. Epidemiol. 32, 377–389. 10.1007/s10654-017-0255-x 28527048 PMC5506233

[B12] Calder P. C. (2015). Marine omega-3 fatty acids and inflammatory processes: effects, mechanisms and clinical relevance. Biochimica biophysica acta 1851, 469–484. 10.1016/j.bbalip.2014.08.010 25149823

[B13] Calder P. C. (2017). Omega-3 fatty acids and inflammatory processes: from molecules to man. Biochem. Soc. Trans. 45, 1105–1115. 10.1042/BST20160474 28900017

[B14] Castañeyra-Ruiz L. González-Marrero I. Carmona-Calero E. M. Abreu-Gonzalez P. Lecuona M. Brage L. (2016). Cerebrospinal fluid levels of tumor necrosis factor alpha and aquaporin 1 in patients with mild cognitive impairment and idiopathic normal pressure hydrocephalus. Clin. Neurol. Neurosurg. 146, 76–81. 10.1016/j.clineuro.2016.04.025 27155076

[B15] Chang C. C. Asada H. Mimura T. Suzuki S. (2009). A prospective study of cerebral blood flow and cerebrovascular reactivity to acetazolamide in 162 patients with idiopathic normal-pressure hydrocephalus. J. Neurosurg. 111, 610–617. 10.3171/2008.10.17676 19284245

[B16] Chen J. Sun B. Zhang D. (2019). Association of dietary n3 and n6 fatty acids intake with hypertension: NHANES 2007-2014. Nutrients 11, 1232. 10.3390/nu11061232 31151215 PMC6627798

[B17] Davies N. M. Holmes M. V. Davey Smith G. (2018). Reading Mendelian randomisation studies: a guide, glossary, and checklist for clinicians. BMJ Clin. Res. ed.) 362, k601. 10.1136/bmj.k601 PMC6041728 30002074

[B18] Dyall S. C. (2017). Interplay between n-3 and n-6 long-chain polyunsaturated fatty acids and the endocannabinoid system in brain protection and repair. Lipids 52, 885–900. 10.1007/s11745-017-4292-8 28875399 PMC5656721

[B19] Dyall S. C. Balas L. Bazan N. G. Brenna J. T. Chiang N. da Costa Souza F. (2022). Polyunsaturated fatty acids and fatty acid-derived lipid mediators: recent advances in the understanding of their biosynthesis, structures, and functions. Prog. lipid Res. 86, 101165. 10.1016/j.plipres.2022.101165 35508275 PMC9346631

[B20] Freeman G. Cowling B. J. Schooling C. M. (2013). Power and sample size calculations for Mendelian randomization studies using one genetic instrument. Int. J. Epidemiol. 42, 1157–1163. 10.1093/ije/dyt110 23934314

[B21] Guan W. Steffen B. T. Lemaitre R. N. Wu J. H. Y. Tanaka T. Manichaikul A. (2014). Genome-wide association study of plasma N6 polyunsaturated fatty acids within the cohorts for heart and aging research in genomic epidemiology consortium. Circ. Cardiovasc. Genet. 7, 321–331. 10.1161/CIRCGENETICS.113.000208 24823311 PMC4123862

[B22] Hakim S. Adams R. D. (1965). The special clinical problem of symptomatic hydrocephalus with normal cerebrospinal fluid pressure. Observations on cerebrospinal fluid hydrodynamics. J. Neurol. Sci. 2, 307–327. 10.1016/0022-510x(65)90016-x 5889177

[B23] Harris W. S. Bulchandani D. (2006). Why do omega-3 fatty acids lower serum triglycerides? Curr. Opin. Lipidol. 17, 387–393. 10.1097/01.mol.0000236363.63840.16 16832161

[B24] He K. Liu K. Daviglus M. L. Jenny N. S. Mayer-Davis E. Jiang R. (2009). Associations of dietary long-chain n-3 polyunsaturated fatty acids and fish with biomarkers of inflammation and endothelial activation (from the Multi-Ethnic Study of Atherosclerosis [MESA]). Am. J. Cardiol. 103, 1238–1243. 10.1016/j.amjcard.2009.01.016 19406265 PMC2697819

[B25] Hoshi T. Wissuwa B. Tian Y. Tajima N. Xu R. Bauer M. (2013). Omega-3 fatty acids lower blood pressure by directly activating large-conductance Ca^2^⁺-dependent K⁺ channels. Proc. Natl. Acad. Sci. U. S. A. 110, 4816–4821. 10.1073/pnas.1221997110 23487785 PMC3607063

[B26] Huang W. Bartosch A. M. Xiao H. Maji S. Youth E. H. H. Flowers X. (2021). An immune response characterizes early Alzheimer's disease pathology and subjective cognitive impairment in hydrocephalus biopsies. Nat. Commun. 12, 5659. 10.1038/s41467-021-25902-y 34580300 PMC8476497

[B27] Israelsson H. Carlberg B. Wikkelsö C. Laurell K. Kahlon B. Leijon G. (2017). Vascular risk factors in INPH: a prospective case-control study (the INPH-CRasH study). Neurology 88, 577–585. 10.1212/WNL.0000000000003583 28062721 PMC5304464

[B28] Israelsson H. Larsson J. Eklund A. Malm J. (2020). Risk factors, comorbidities, quality of life, and complications after surgery in idiopathic normal pressure hydrocephalus: review of the INPH-CRasH study. Neurosurg. Focus 49, E8. 10.3171/2020.7.FOCUS20466 33002861

[B29] Jaraj D. Rabiei K. Marlow T. Jensen C. Skoog I. Wikkelsø C. (2014). Prevalence of idiopathic normal-pressure hydrocephalus. Neurology 82, 1449–1454. 10.1212/WNL.0000000000000342 24682964 PMC4001197

[B30] Kapoor B. Kapoor D. Gautam S. Singh R. Bhardwaj S. (2021). Dietary polyunsaturated fatty acids (PUFAs): uses and potential health benefits. Curr. Nutr. Rep. 10, 232–242. 10.1007/s13668-021-00363-3 34255301

[B31] Kristensen B. Malm J. Fagerland M. Hietala S. O. Johansson B. Ekstedt J. (1996). Regional cerebral blood flow, white matter abnormalities, and cerebrospinal fluid hydrodynamics in patients with idiopathic adult hydrocephalus syndrome. J. neurology, Neurosurg. psychiatry 60, 282–288. 10.1136/jnnp.60.3.282 PMC1073850 8609504

[B32] Kurki M. I. Karjalainen J. Palta P. Sipilä T. P. Kristiansson K. Donner K. M. (2023). FinnGen provides genetic insights from a well-phenotyped isolated population. Nature 613, 508–518. 10.1038/s41586-022-05473-8 36653562 PMC9849126

[B33] Lawlor D. A. Harbord R. M. Sterne J. A. Timpson N. Davey Smith G. (2008). Mendelian randomization: using genes as instruments for making causal inferences in epidemiology. Statistics Med. 27, 1133–1163. 10.1002/sim.3034 17886233

[B34] Lechner K. von Schacky C. McKenzie A. L. Worm N. Nixdorff U. Lechner B. (2020). Lifestyle factors and high-risk atherosclerosis: pathways and mechanisms beyond traditional risk factors. Eur. J. Prev. Cardiol. 27, 394–406. 10.1177/2047487319869400 31408370 PMC7065445

[B35] Lemaitre R. N. Tanaka T. Tang W. Manichaikul A. Foy M. Kabagambe E. K. (2011). Genetic loci associated with plasma phospholipid n-3 fatty acids: a meta-analysis of genome-wide association studies from the CHARGE Consortium. PLoS Genet. 7, e1002193. 10.1371/journal.pgen.1002193 21829377 PMC3145614

[B36] Li H. Liu C. Tai H. Wei Y. Shen T. Yang Q. (2023). Comparison of cerebrospinal fluid space between probable normal pressure hydrocephalus and Alzheimer's disease. Front. Aging Neurosci. 15, 1241237. 10.3389/fnagi.2023.1241237 37693646 PMC10484096

[B37] Li X. Bi X. Wang S. Zhang Z. Li F. Zhao A. Z. (2019). Therapeutic potential of ω-3 polyunsaturated fatty acids in human autoimmune diseases. Front. Immunol. 10, 2241. 10.3389/fimmu.2019.02241 31611873 PMC6776881

[B38] Lin Z. Pan I. Pan W. (2022). A practical problem with Egger regression in Mendelian randomization. PLoS Genet. 18, e1010166. 10.1371/journal.pgen.1010166 35507585 PMC9109933

[B39] Liu X. Hao J. Yao E. Cao J. Zheng X. Yao D. (2020). Polyunsaturated fatty acid supplement alleviates depression-incident cognitive dysfunction by protecting the cerebrovascular and glymphatic systems. Brain, Behav. Immun. 89, 357–370. 10.1016/j.bbi.2020.07.022 32717402

[B40] Marion-Letellier R. Savoye G. Ghosh S. (2015). Polyunsaturated fatty acids and inflammation. IUBMB life 67, 659–667. 10.1002/iub.1428 26397837

[B41] McGirr A. Vila-Rodriguez F. Cusimano M. D. (2015). “Chapter 10 - normal pressure hydrocephalus: etiology, diagnosis, treatment, and putative nutritional and lifestyle risk factors,” in Diet and nutrition in dementia and cognitive decline. Editors Martin C. R. Preedy V. R. (San Diego: Academic Press), 101–112.

[B42] Preuss M. Hoffmann K. T. Reiss-Zimmermann M. Hirsch W. Merkenschlager A. Meixensberger J. (2013). Updated physiology and pathophysiology of CSF circulation--the pulsatile vector theory. Child's Nerv. Syst. ChNS official J. Int. Soc. Pediatr. Neurosurg. 29, 1811–1825. 10.1007/s00381-013-2219-0 23832074

[B43] Russo G. L. (2009). Dietary n-6 and n-3 polyunsaturated fatty acids: from biochemistry to clinical implications in cardiovascular prevention. Biochem. Pharmacol. 77, 937–946. 10.1016/j.bcp.2008.10.020 19022225

[B44] Schini V. B. Durante W. Catovsky S. Vanhoutte P. M. (1993). Eicosapentaenoic acid potentiates the production of nitric oxide evoked by interleukin-1 beta in cultured vascular smooth muscle cells. J. Vasc. Res. 30, 209–217. 10.1159/000158996 8357951

[B45] Smith G. D. Ebrahim S. (2003). Mendelian randomization': can genetic epidemiology contribute to understanding environmental determinants of disease? Int. J. Epidemiol. 32, 1–22. 10.1093/ije/dyg070 12689998

[B46] Smith G. D. Ebrahim S. (2004). Mendelian randomization: prospects, potentials, and limitations. Int. J. Epidemiol. 33, 30–42. 10.1093/ije/dyh132 15075143

[B47] Sosvorova L. Vcelak J. Mohapl M. Vitku J. Bicikova M. Hampl R. (2014). Selected pro- and anti-inflammatory cytokines in cerebrospinal fluid in normal pressure hydrocephalus. Neuro Endocrinol. Lett. 35, 586–593. 25617881

[B48] Tan C. Wang X. Wang Y. Wang C. Tang Z. Zhang Z. (2021). The pathogenesis based on the glymphatic system, diagnosis, and treatment of idiopathic normal pressure hydrocephalus. Clin. interventions aging 16, 139–153. 10.2147/CIA.S290709 PMC7815082 33488070

[B49] Verbanck M. Chen C. Y. Neale B. Do R. (2018). Detection of widespread horizontal pleiotropy in causal relationships inferred from Mendelian randomization between complex traits and diseases. Nat. Genet. 50, 693–698. 10.1038/s41588-018-0099-7 29686387 PMC6083837

[B50] Wang Z. Zhang Y. Hu F. Ding J. Wang X. (2020). Pathogenesis and pathophysiology of idiopathic normal pressure hydrocephalus. CNS Neurosci. Ther. 26, 1230–1240. 10.1111/cns.13526 33242372 PMC7702234

[B51] Yavorska O. O. Burgess S. (2017). MendelianRandomization: an R package for performing Mendelian randomization analyses using summarized data. Int. J. Epidemiol. 46, 1734–1739. 10.1093/ije/dyx034 28398548 PMC5510723

[B52] Zheng J. Erzurumluoglu A. M. Elsworth B. L. Kemp J. P. Howe L. Haycock P. C. (2017). LD Hub: a centralized database and web interface to perform LD score regression that maximizes the potential of summary level GWAS data for SNP heritability and genetic correlation analysis. Bioinforma. Oxf. Engl. 33, 272–279. 10.1093/bioinformatics/btw613 PMC5542030 27663502

