# Supplementary material for: Positive association between omega-3/6 polyunsaturated fatty acids and idiopathic normal pressure hydrocephalus: a mendelian randomization study
Source: Front Genet. 2023 Dec 19;14:1269494. doi: 10.3389/fgene.2023.1269494 (PMC10762850; doi:10.3389/fgene.2023.1269494)
Supplement: Supplementary file 1 [file DataSheet1.PDF]

Positive association between omega-3/6 polyunsaturated fatty acids and idiopathic normal pressure hydrocephalus: a mendelian randomization study

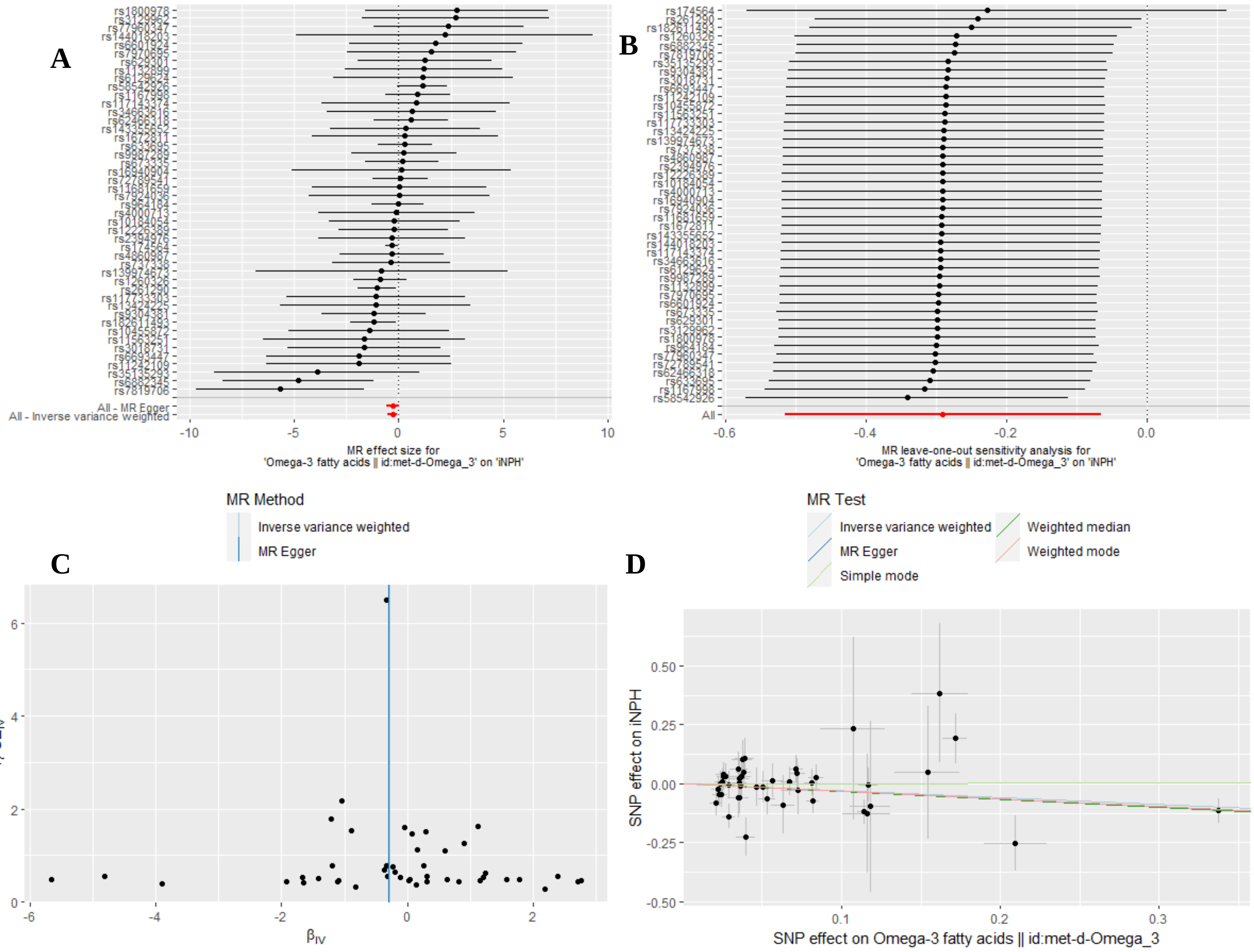

Supplementary Figure 1. Forest plot(A), leave-one-out plot(B), funnel plot(C), scatter plot(D) of genetic association between omega-3 PUFAs and iNPH.

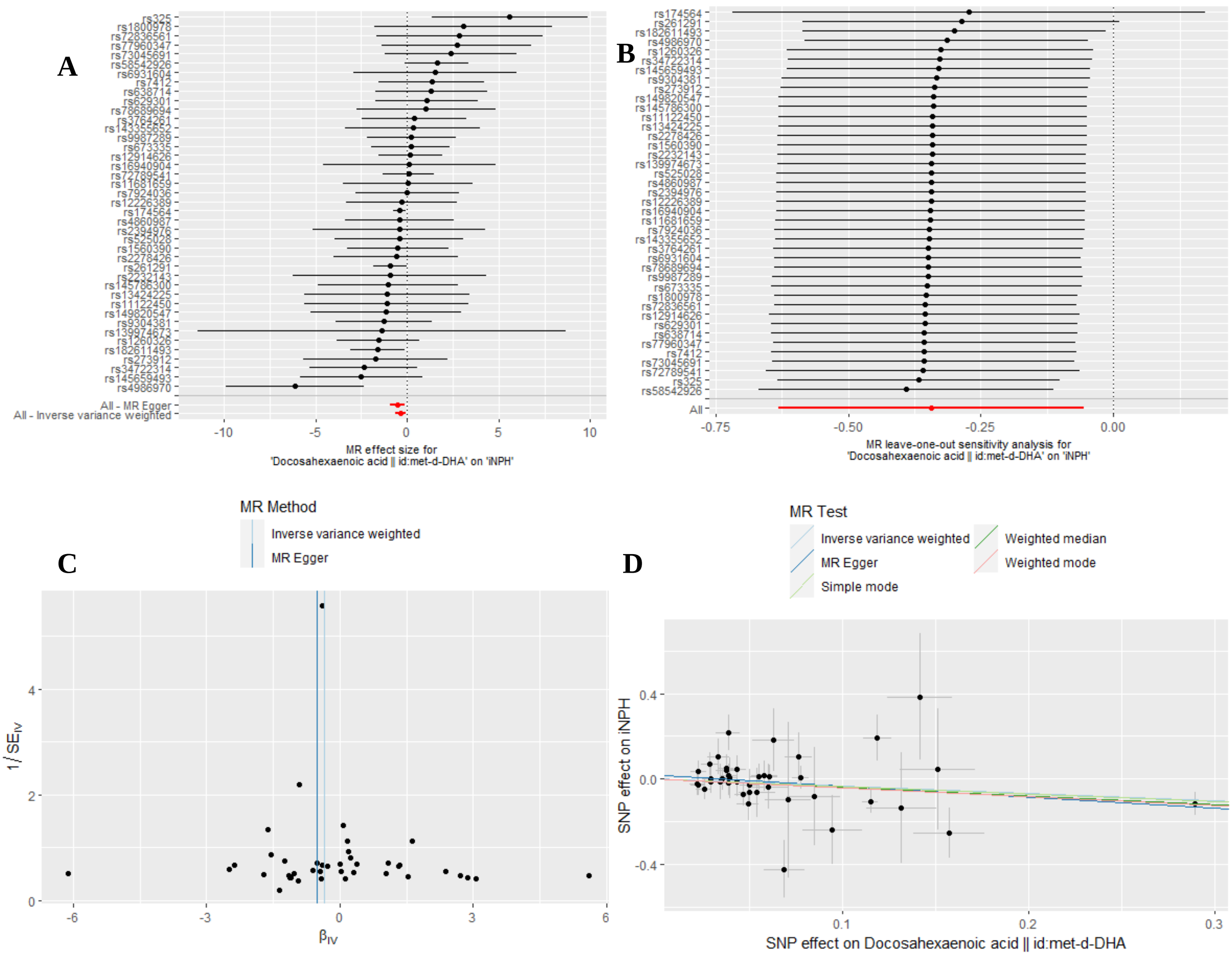

Supplementary Figure 2. Forest plot(A), leave-one-out plot(B), funnel plot(C), scatter plot(D) of genetic association between DHA and iNPH.

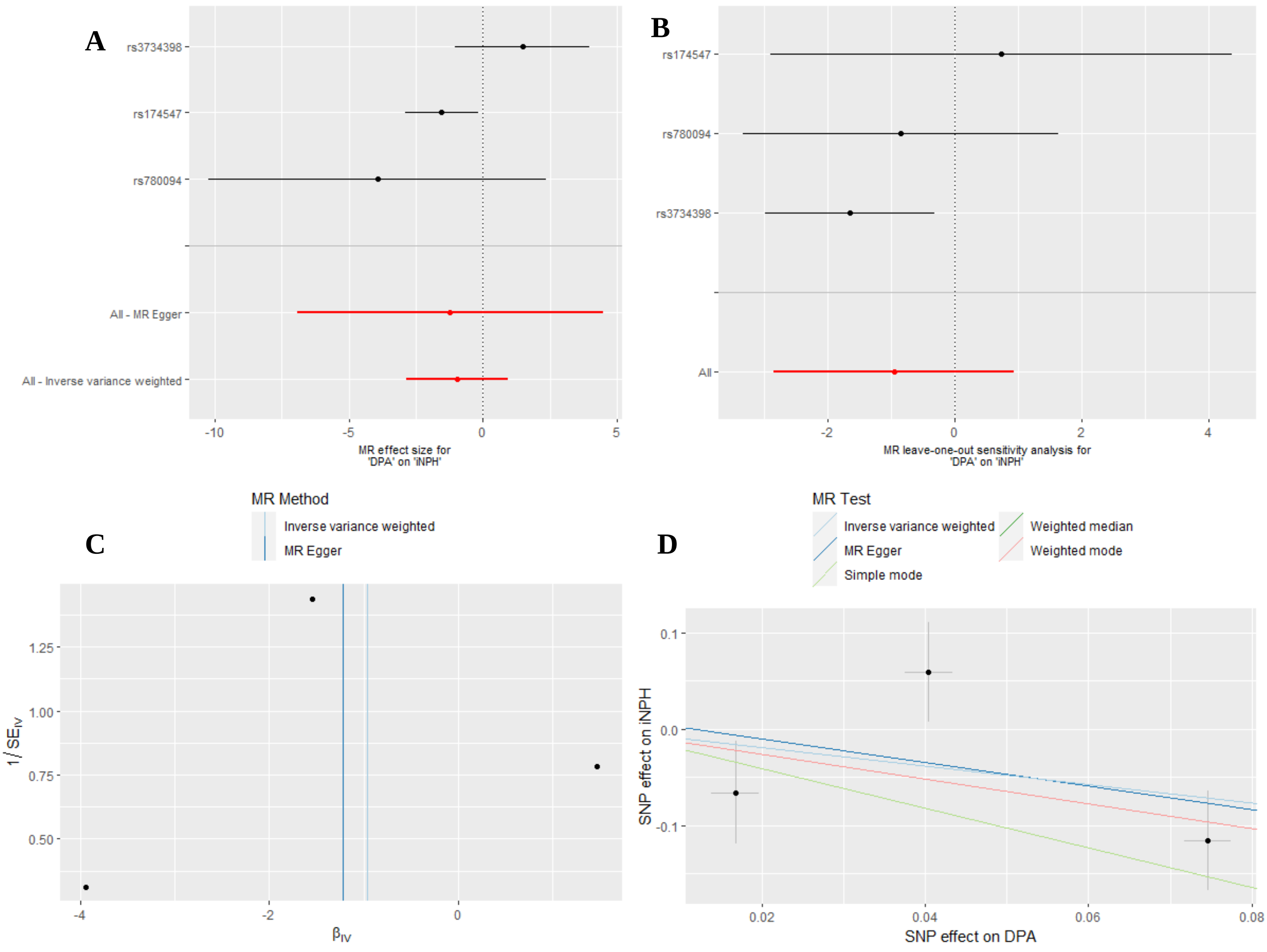

Supplementary Figure 3. Forest plot(A), leave-one-out plot(B), funnel plot(C), scatter plot(D) of genetic association between DPA and iNPH.

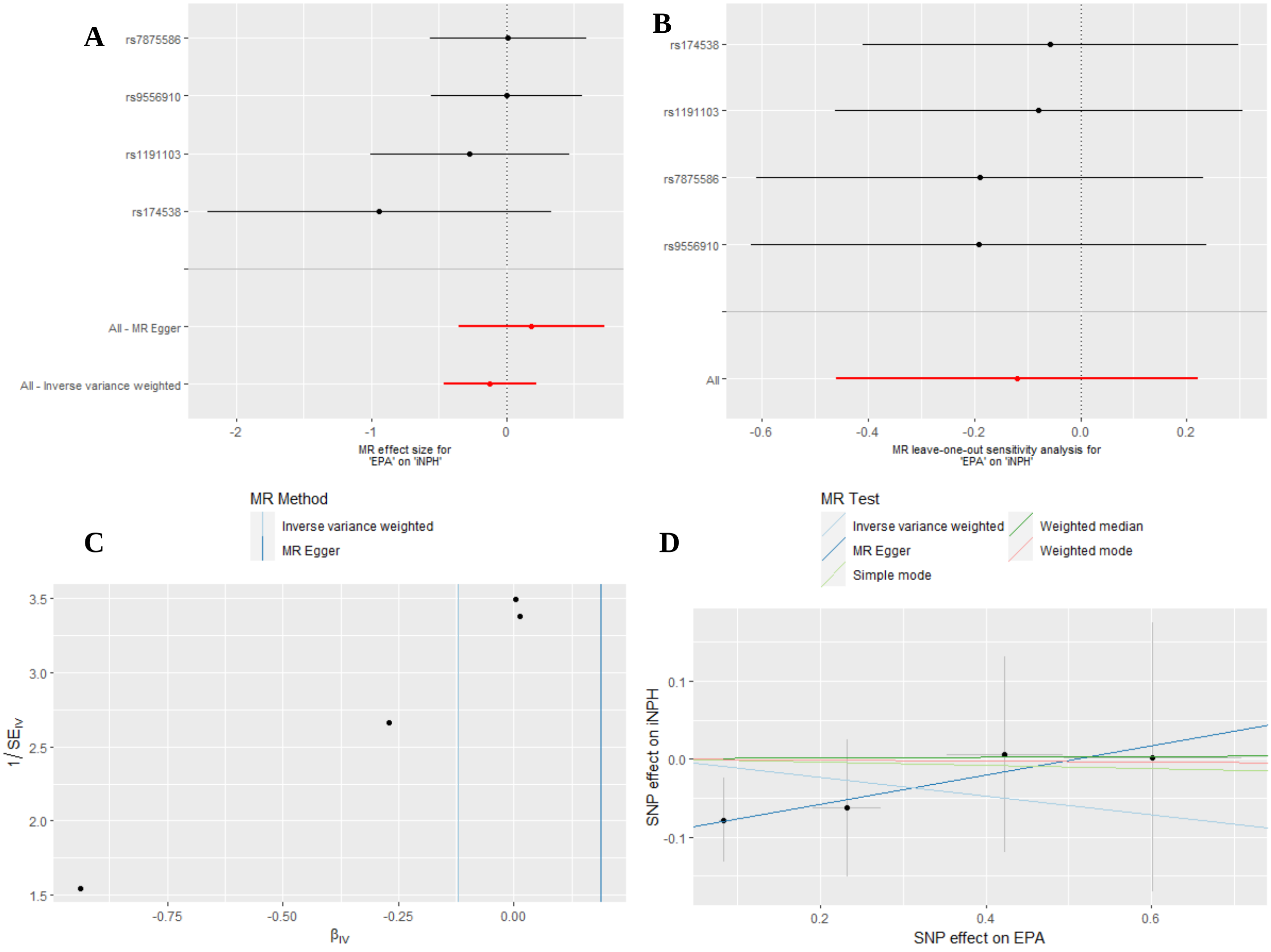

Supplementary Figure 4. Forest plot(A), leave-one-out plot(B), funnel plot(C), scatter plot(D) of genetic association between EPA and iNPH.

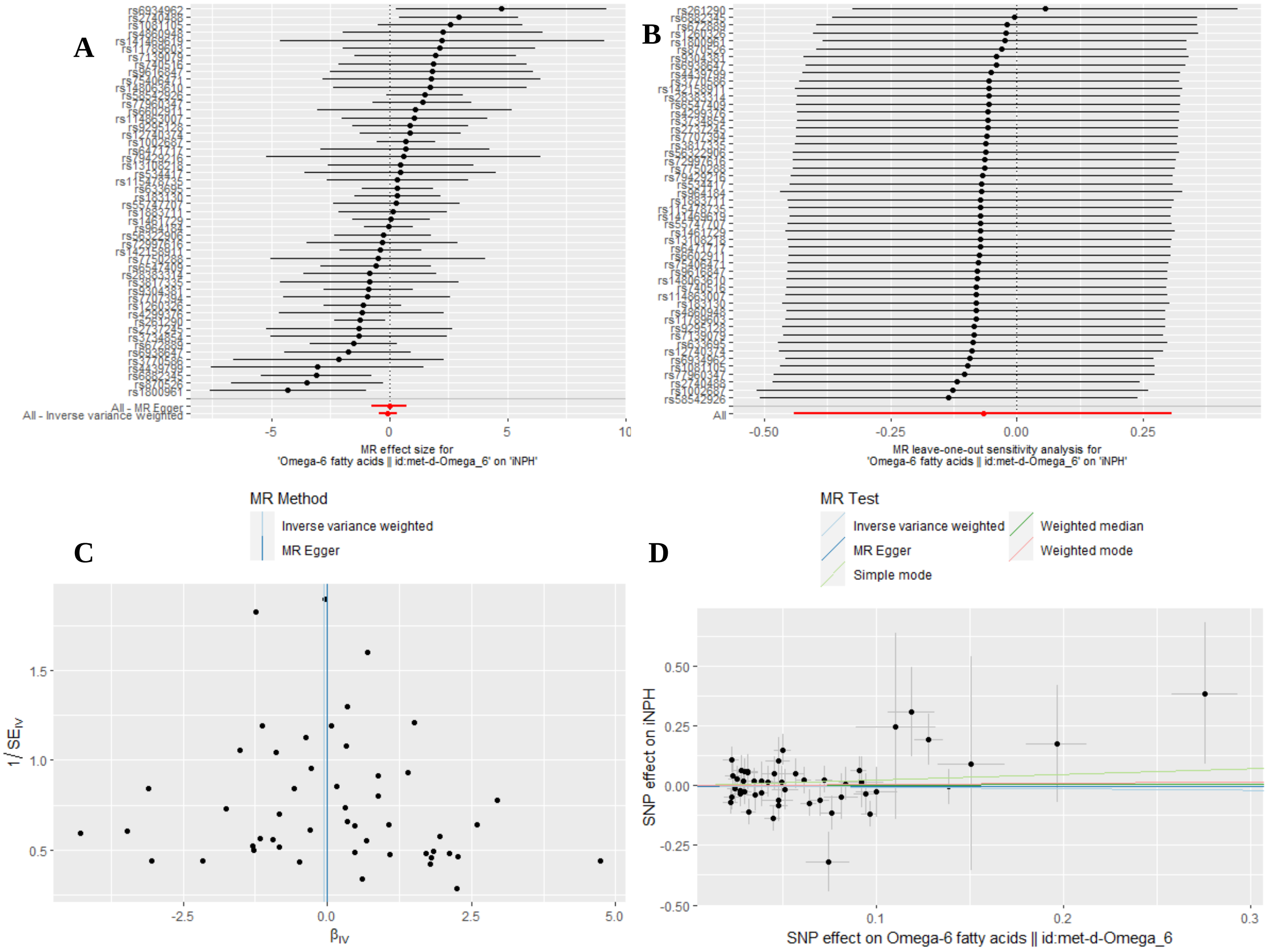

Supplementary Figure 5. Forest plot(A), leave-one-out plot(B), funnel plot(C), scatter plot(D) of genetic association between omega-6 PUFAs and iNPH.

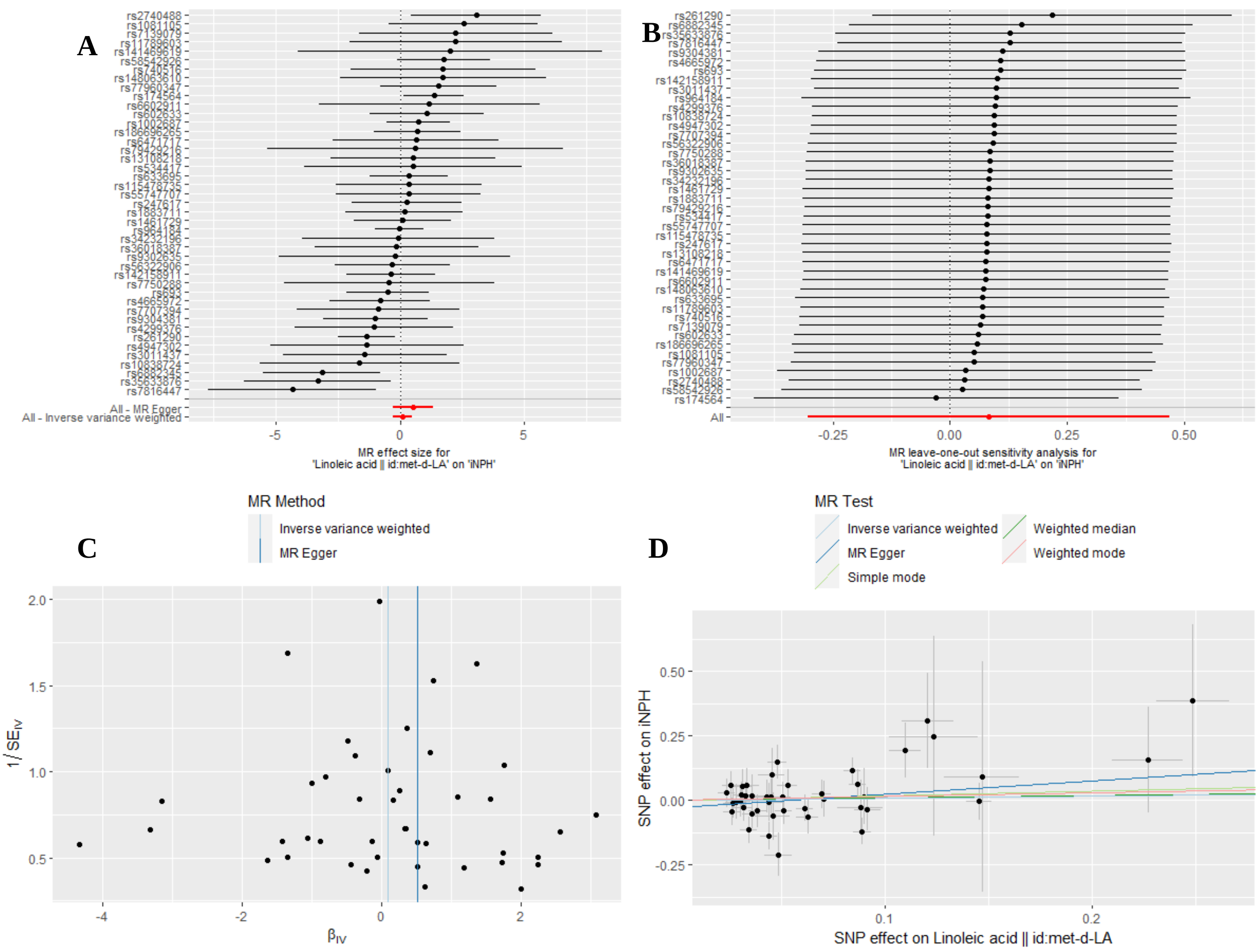

Supplementary Figure 6. Forest plot(A), leave-one-out plot(B), funnel plot(C), scatter plot(D) of genetic association between LA and iNPH.

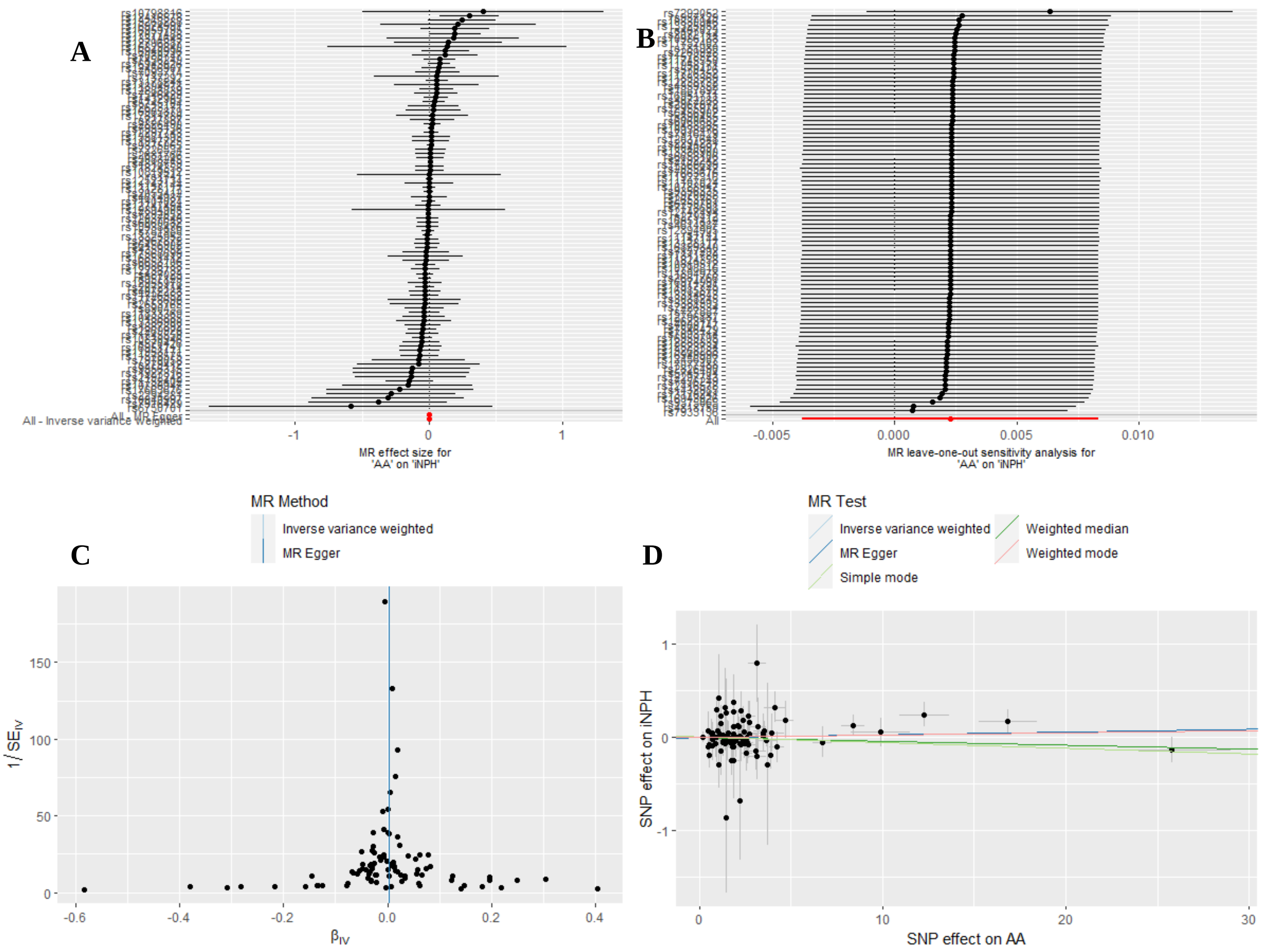

Supplementary Figure 7. Forest plot(A), leave-one-out plot(B), funnel plot(C), scatter plot(D) of genetic association between AA and iNPH.
